# New Spinal Shortening Technique for Tethered Cord Syndrome: A Technical Note

**DOI:** 10.3390/medicina60010020

**Published:** 2023-12-22

**Authors:** Masato Tanaka, Sumeet Sonawane, Shinya Arataki, Yoshihiro Fujiwara, Takuya Taoka, Koji Uotani, Yoshiaki Oda, Kensuke Shinohara

**Affiliations:** 1 Department of Orthopaedic Surgery, Okayama Rosai Hospital, Okayama 702-8055, Japan; drsumeet166@gmail.com (S.S.); araoyc@gmail.com (S.A.); fujiwarayoshihiro2004@yahoo.co.jp (Y.F.); taokatakuya@gmail.com (T.T.); 2 Department of Orthopaedic Surgery, Okayama University Hospital, Okayama 700-8558, Japan; coji.uo@gmail.com (K.U.); odaaaaaaamn@yahoo.co.jp (Y.O.); joker1011ks@yahoo.co.jp (K.S.)

**Keywords:** tethered cord syndrome, navigation, spinal shortening osteotomy

## Abstract

*Background and Objectives*: To present a new spinal shortening technique for tethered cord syndrome. Tethered cord syndrome (TCS) is a debilitating condition leading to progressive neurological decline. Surgical detethering for TCS is the gold standard of treatment. However, symptomatic retethering of TCS has been reported in 5%–50% of patients after initial release. To solve this problem, posterior spinal shortening osteotomy has been reported. This technique has risks of massive blood loss and neurological deterioration. The authors hereby report a new safe spinal shortening technique for tethered cord syndrome. *Materials and Methods:* A 31-year-old man with gait disturbance was referred to our hospital. After the delivery of treatment, he underwent surgical untethering of the spinal cord in another hospital. He had hyperreflexia of the Achilles tendon reflex and bilateral muscle weakness of the legs (MMT 3-4). He also had urinary and bowel incontinence, and total sensory loss below L5. An anteroposterior lumbar radiogram indicated partial laminectomy of L3 and L4. Lumbar MRI showed retethering of spinal cord. *Results:* The patient underwent a new spinal shortening technique for tethered cord syndrome under the guidance of O-arm navigation. First, from the anterior approach, disectomy from T12 to L3 was performed. Second, from the posterior approach, Ponte osteotomy was performed from T12 to L3, shortening the spinal column by 15 mm. The patient was successfully treated surgically. Postoperative lumbar MRI showed that the tension of the spinal cord was released. Manual muscle testing results and the sensory function of the left leg had recovered almost fully upon final follow-up at one year. *Conclusions*: A retethered spinal cord after initial untethering is difficult to treat. This new spinal shortening technique can represent another good option to release the tension of the spinal cord.

## 1. Introduction

Tethered cord syndrome (TCS) is defined as the traction of the lower end of the spinal cord by a thickened filum terminale or spinal lipoma [1,2]. The symptoms of TCS are low back pain; lower-extremity motor and sensory deficits; urinary and bowel incontinence; and sexual dysfunction [3]. If the symptoms are severe and progressive, surgical intervention is indicated [4]. The aim of surgery is to release the spinal cord and neural elements from tension to restore neurological function and prevent further deterioration [5]. The gold standard for TCS surgery is untethering of the spinal cord [6,7]. The results of detethering are relatively good, providing improvement in urinary symptoms in 40–60% of cases, an improvement in motor disturbance in 40–70% of cases, and pain improvement in 80–90% of cases [5]. However, recurrent or retethering of the spinal cord has been reported in more than 25% of cases [6,7,8].

Because of the high prevalence of complications such as retethering, arachnoid adhesion, cerebral fluid leakage, psuedomeningocele, and wound problems [9,10], in 2009, Miyakoshi et al. reported a spine-shortening vertebral osteotomy (SVO) for TCS to relieve the longitudinal tension placed on the tethered neural elements without violating the dura [11]. After their reports, many authors reported excellent results for this technique [5,12]. However, the disadvantages of SVO include massive blood loss and some risk of neurological deterioration [13]. The authors hereby present a novel technique for shortening the spinal column without such risks.

This study was approved by the ethics committee of our institute (No. 434). Necessary consent was obtained from the patient.

## 2. Case 1: 31-Year-Old Male, Tethered Cord Syndrome

### 2.1. Patient History

A 31-year-old man with gait disturbance was referred to our hospital. After the delivery of treatment, he underwent surgical untethering of the spinal cord (due to possible lipoma) in another hospital. 

### 2.2. Physical Examination

The patient could walk without any support. In the examination, he had hyperreflexia of the Achilles tendon reflex and bilateral muscle weakness of the legs (manual muscle test: MMT, right left, tibialis anterior 4 4, extensor halluces longus 4 3, flexor halluces longus 4 2). He also had urinary and bowel incontinence, and total bilateral sensory loss below L5. 

### 2.3. Preoperative Imaging

**Figure 1:**
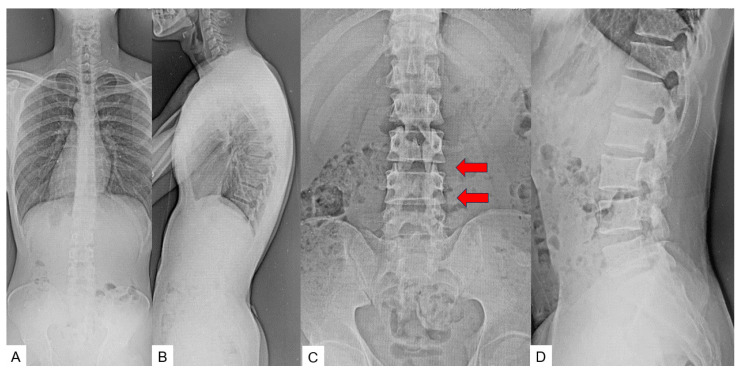
Preoperative radiograms. (**A**) Full spine standing postero-anterior radiogram. (**B**) Full spine standing lateral radiogram. (**C**) Lumbar antero-posterior radiogram. (**D**) Lumbar lateral radiogram. L3.4 displays partial laminectomy because of the previous detethering surgery (red arrows).

**Figure 2:**
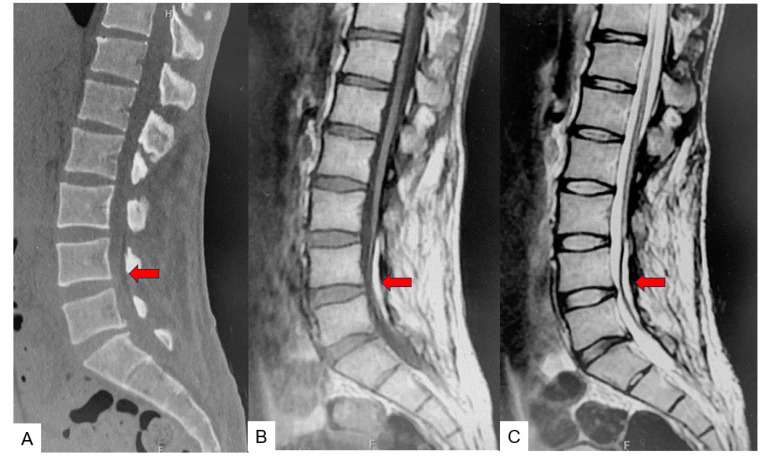
Preoperative CT and MR imaging. (**A**) Mid sagittal 3D reconstruction CT. (**B**) T1 weighted mid-sagittal MR imaging. (**C**) T2 weighted mid-sagittal MR imaging. A lipoma is attached to the spinal cord and retethering is observed (red arrows).

Preoperative anteroposterior lumbar radiogram indicates partial laminectomy of L3 and L4 (Figure 1). Preoperative lumbar CT showed a small spinous process from L2 to S1. Preoperative MRI indicated retethering or incomplete untethering of the spinal cord. In the L4.5 spinal canal, a lipoma was attached to the spinal cord and retethering was observed (Figure 2).

### 2.4. Surgery

#### 2.4.1. Anterior Discectomy

**Figure 3:**
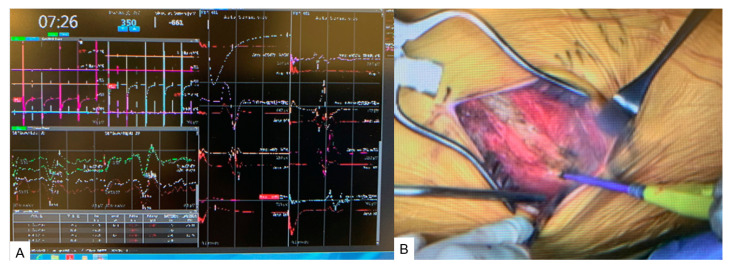
Neuromonitoring and anterior approach. (**A**) Neuromonitoring. (**B**) The 11th rib resection.

The patient underwent a new spinal shortening technique for tethered cord syndrome under the guidance of O-arm navigation (Figure 3A). The surgery was performed under intravenous anesthesia with propofol because of neuromonitoring. The first step was the anterior approach. The patient was placed in a right decubitus position on a full carbon table. The percutaneous reference frame was inserted into the left sacroiliac joint. Then, an O-arm scan was performed and 3D images were transferred to the navigation. The left 11th rib was resected with a 5cm oblique skin incision (Figure 3B). 

**Figure 4:**
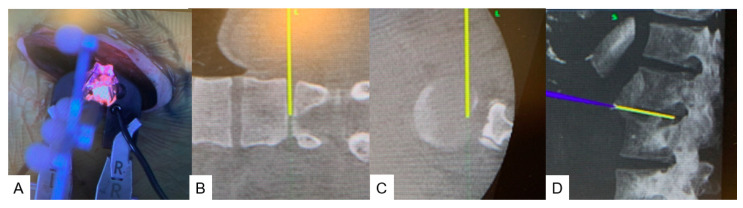
Navigated pointer. (**A**) Intraoperative image. (**B**) Navigation monitor (coronal). (**C**) Navigation monitor (Axial). (**D**) Navigation monitor (3D).

**Figure 5:**
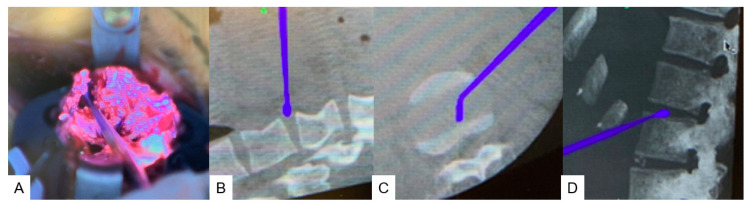
Navigated curette. (**A**) Intraoperative image. (**B**) Navigation monitor (coronal). (**C**) Navigation monitor (axial). (**D**) Navigation monitor (3D).

**Figure 6:**
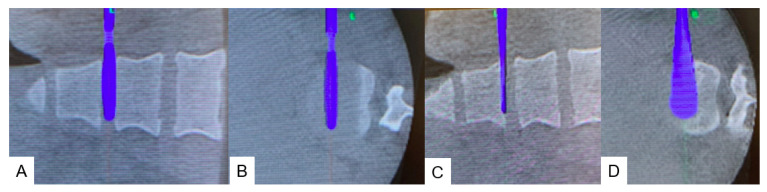
Navigated shaver and navigated Cobb. (**A**) Navigated shaver (coronal). (**B**) Navigated shaver (axial). (**C**) Navigated Cobb (coronal). (**D**) Navigated Cobb (axial).

The disc level as identified with a navigated probe (Figure 4). Discectomy was performed with a navigated curette (Figure 5), a navigated shaver (Figure 6A,B), and a navigated Cobb (Figure 6C,D). After total T11-L3 discectomies were completed, the wound was closed in layers.

#### 2.4.2. Posterior Osteotomy

**Figure 7:**
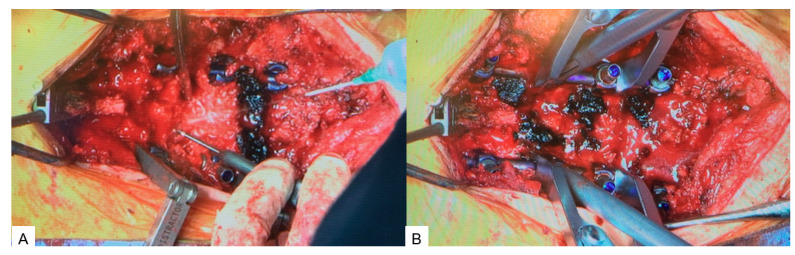
Posterior surgery. (**A**) Ponte osteotomy. (**B**) Spinal shortening with a compressor.

**Figure 8:**
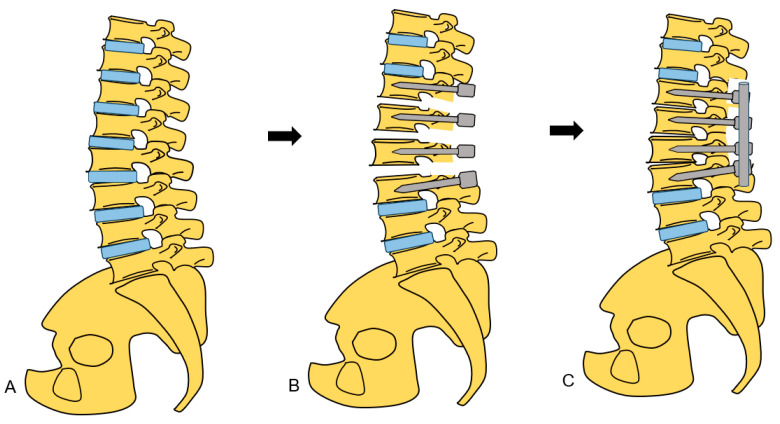
Schema of new spinal shortening osteotomy. (**A**) Preoperative image, (**B**) After disc removal, (**C**) After spinal shortening.

On the same day, the patient was placed in the prone position on a full carbon table. Another O-arm scan was performed. Then, posterior elements were exposed with a longitudinal incision. Pedicle screws from T11 to L3 were inserted under navigation guidance. The Ponte osteotomy and posterior anulus were resected from T11/12 to L2/3 (Figure 7A). Finally, compression force was applied to each disc level with a compressor (Figure 7B). After the whole procedure was completed, the spinal column was shortened by 15 mm (Figure 8). During the procedure, an antifibrinolytic drug and a cell saver were used. However, blood transfusion was not necessary.

The patient was successfully treated surgically. The surgical time was 4 h and 10 min and the estimated blood loss was 775 mL. The patient experienced no postoperative complications such as dural tear, surgical site infection, or neurological compromise.

### 2.5. Postoperative Images

**Figure 9:**
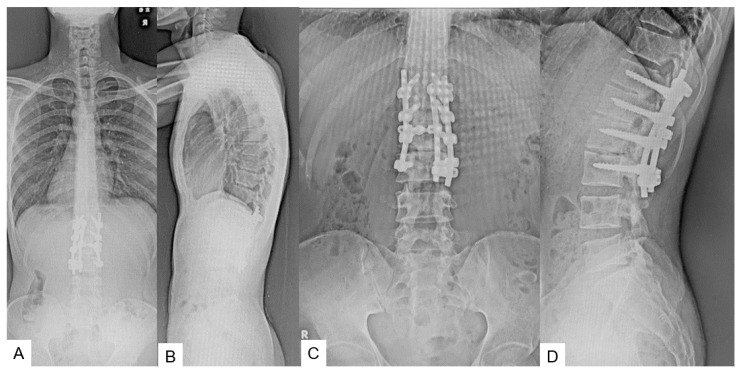
Postoperative images. (**A**) Full spine standing postero-anterior radiogram. (**B**) Full spine standing lateral radiogram. (**C**) Lumar antero-posterior radiogram. (**D**) Lumbar lateral radiogram. The spinal column was shortened 15 mm.

The postoperative radiograms showed good alignment, and the shortening of the spinal column was 15 mm. The bony resection area was adequate (Figure 9). 

### 2.6. One Year Follow-Up

**Figure 10:**
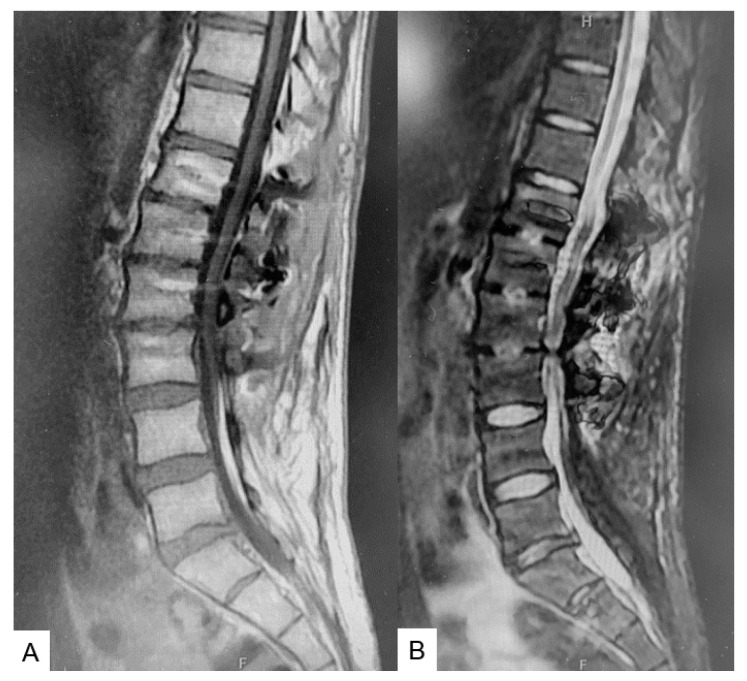
Follow-up images. (**A**) Mid sagittal T1-weighted MR imaging. (**B**) Mid sagittal T2-weighted MR imaging.

The patient had recovered almost fully at six months follow-up. The syringomyelia showed a decrease in morphology (Figure 10). Manual muscle testing had slightly recovered and sensory function of the left leg had recovered almost fully upon final follow-up at one year.

## 3. Case 2: 33-Year-Old Male, Tethered Cord Syndrome, Conventional Technique

**Figure 11:**
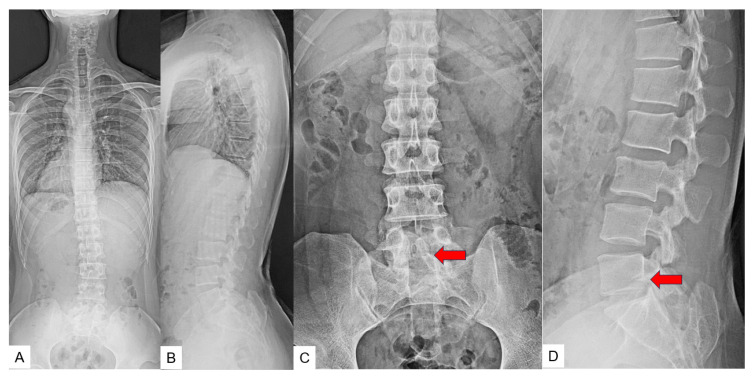
Preoperative radiograms. (**A**) Full spine standing postero-anterior radiogram. (**B**) Full spine standing lateral radiogram. (**C**) Lumar antero-posterior radiogram. (**D**) Lumbar lateral radiogram. L3.4 displays partial laminectomy because of the previous detethering surgery (red arrows).

**Figure 12:**
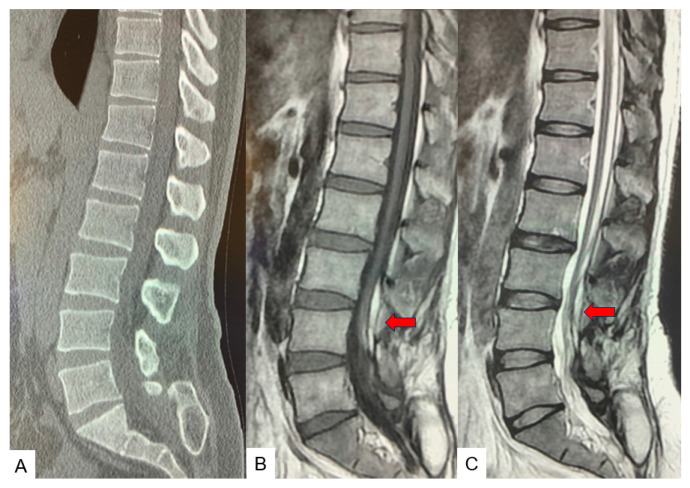
Preoperative CT and MR imaging. (**A**) Mid sagittal 3D reconstruction CT. (**B**) T1 weighted mid-sagittal MR imaging. (**C**) T2 weighted mid-sagittal MR imaging. A lipoma is attached to the spinal cord and retethering is observed (red arrows).

This patient underwent a conventional shortening osteotomy. Preoperative radiograms (Figure 11), CT and MRI (Figure 12) are presented. 

**Figure 13:**
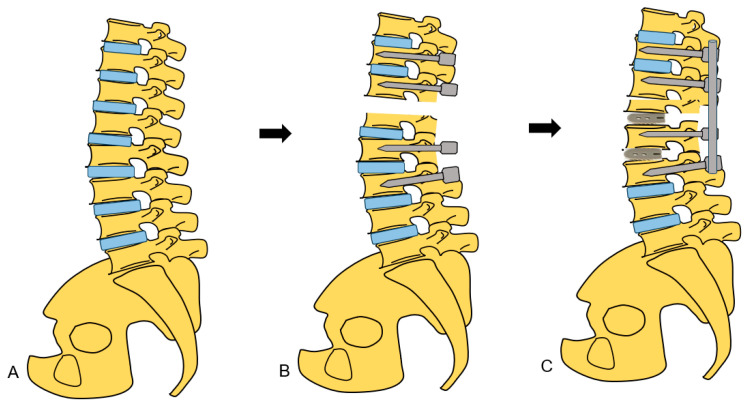
Shema of conventional spinal shortening osteotomy. (**A**) Preoperative image, (**B**) After partial vertebra removal, (**C**) After spinal shortening.

**Figure 14:**
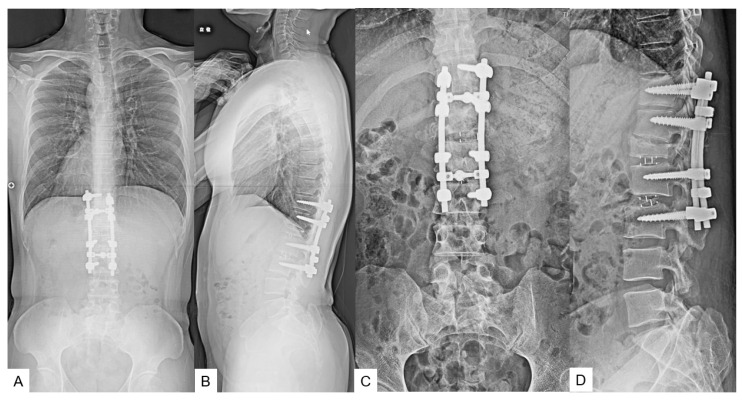
Postoperative images. (**A**) Full spine standing postero-anterior radiogram. (**B**) Full spine standing lateral radiogram. (**C**) Lumar antero-posterior radiogram. (**D**) Lumbar lateral radiogram. The spinal column was shortened by 20 mm.

The conventional spinal column shortening osteotomy was performed (Figure 13 and Figure 14). The surgical time was 6 h and 57 min and the estimated blood loss was 2370 mL. 

**Figure 15:**
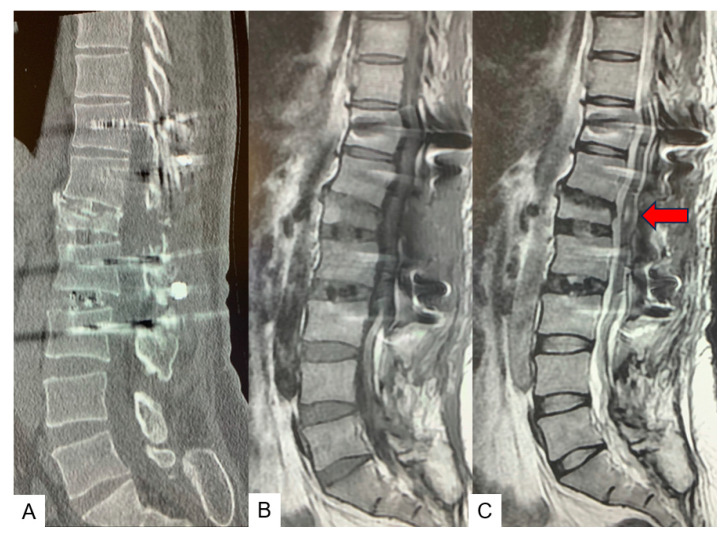
Follow-up images. (**A**) Mid sagittal reconstruction CT, (**B**) Mid sagittal T1-weighted MR imaging, (**C**) Mid sagittal T2-weighted MR imaging. There is as T2 high area in the spinal cord (red arrow).

After the surgery, the patient’s neurological status was slightly deteriorated. Postoperative CT showed that good spinal shortening was achieved, but spinal cord damage was observed in the osteotomy site (Figure 15). After two years of follow up, the patient’s symptoms were almost entirely alleviated. 

## 4. Discussion

Garceau first described ‘filum terminale syndrome’ in 1953 [14]. Hoffmann, in 1976, first coined the term ‘tethered cord syndrome’, and described it as occurring due to traction of the spinal cord due to a thick filum terminale [15]. Later, Yamada further included conditions like meningomyelocele, lipomeningomyelocele, diastematomyelia, intradural lipoma, and a dermoid sinus in tethered cord syndrome [16]. It has been postulated that disproportionate growth of the vertebral column and spinal cord leads to nerve root traction and metabolic changes.

The clinical presentation of tethered cord syndrome varies according to the age and etiology. Infants with this syndrome may show lipomas, tufts of hair, nevi, hemangiomas, and dermal sinuses or cutaneous manifestations of spina bifida occulta. Scoliosis or lower limb deformities may be present [2]. High suspicion of this syndrome should be kept in mind if anorectal malformations are present in a child. Toddlers may present with pain, sensory motor deficit, scoliosis, bladder dysfunction, lower limb deformities, and gait disturbance [17].

The trend of treatment for TCS has changed from wait and watch to early intervention in the form of surgical detethering, as the natural history of this disease is of gradual progression [9]. Warder et al. reported sensory improvement in 100% of cases, motor improvement in 67% of cases, bladder and bowel symptom improvement in 75% and 100% of cases, respectively [18]. A systemic review by O’Connor et al. reported that after detethering, sensory deficit improved in 45% of cases, motor deficit improved in 61% of cases, and bladder and bowel dysfunction improved in 45% and 32% of cases, respectively [19]. But detethering surgery is associated with complications like CSF leak, surgical site infection, meningitis. Although major problem is retethering, which can be seen in about 5% to 50% of cases [7,8,20].

Because of these complications, Kokubun et al. [21] described an alternative treatment in the form of spine vertebral shortening osteotomy for TCS, and Miyakoshi [11] later presented their results with this technique. The aim of this procedure is to indirectly reduce traction of neural elements by means of spinal column shortening. However, complications in these procedures can have a prevalence of as high as 40%, which include neurological injury and massive blood loss [13].

In our procedure, we performed anterior O-arm-guided C arm free discectomy followed by posterior osteotomy and fixation. Anterior discectomy is a muscle splitting approach with minimal blood loss and can be performed within a short timeframe with the help of O-arm guidance [22]. Pontes osteotomy and pedicle screw fixation can be performed with precision and in a shorter timeframe with O-arm guidance, which reduces blood loss and the risk of neurological injury [23].

Radiation exposure among surgeons and operating staff is a major concern. Radiation exposure can lead to various health problems like early cataracts, infertility, and cancers [24]. With the use of an O arm, there is no radiation exposure to operating staff and surgeon.

## 5. Conclusions

A retethered spinal cord after initial untethering is difficult to treat. This new spinal shortening technique can become another good option to release the tension of the spinal cord.

## Data Availability

The data presented in this study are available in the article.
